# Electromagnetic Force-Driven Needle-Free in Ovo Injection Device

**DOI:** 10.3390/vetsci9030147

**Published:** 2022-03-21

**Authors:** Ko-Jung Huang, Cheng-Han Li, Ping-Kun Tsai, Chia-Chun Lai, Yu-Ren Kuo, Ming-Kun Hsieh, Ching-Wei Cheng

**Affiliations:** 1 Department of Bio-Industrial Mechatronics Engineering, National Chung Hsing University, Taichung 40227, Taiwan; d103040002@mail.nchu.edu.tw (K.-J.H.); d104040001@mail.nchu.edu.tw (P.-K.T.); d107040003@mail.nchu.edu.tw (C.-C.L.); joe213213@gmail.com (Y.-R.K.); 2 Graduate Institute of Microbiology and Public Health, College of Veterinary Medicine, National Chung Hsing University, Taichung 40227, Taiwan; mhsieh@nchu.edu.tw; 3 Department of Computer Science and Information Engineering, National Taichung University of Science and Technology, Taichung 404, Taiwan

**Keywords:** in ovo, needle-free, embryonic egg, injection device

## Abstract

Needle-free injections are mainly used for administering human or mammalian vaccines or drugs. However, poultry vaccines, in ovo injections to embryos, subcutaneous injections to chickens, and intramuscular injections are administered using needle injections. This article presents a new needle-free in ovo injection device method that uses push-pull solenoids to eject liquid jets, mainly for embryonic eggs of chickens. Furthermore, our study investigated the suitable jet pressures for using this method and the post-injection hatching rates in 18-day-old embryonic eggs. Using this method, we could deliver the liquid to the allantoic and amniotic cavities or the muscle tissue through the egg membrane of the air chamber using a jet pressure of ~6–7 MPa or ~8 MPa. After injecting 0.25 mL of 0.9% saline into 18-day-old Lohmann breed layer embryonic eggs and specific pathogen-free (SPF) embryonic eggs at a jet pressure of ~7 MPa, we observed hatching rates of 98.3% and 85.7%, respectively. This study’s electromagnetic needle-free in ovo injection device can apply vaccine or nutrient solution injection for embryo eggs and serve as a reference for future studies on needle-free in ovo injection automation systems, jet pressure control, and injection pretreatment processes.

## 1. Introduction

The global commercial poultry farming industry is gradually growing, and the level of poultry farming technology is also improving. Moreover, the global population’s daily demand for animal protein is increasing [1], and poultry eggs and chickens are among the most commonly consumed protein sources in daily life. Chickens are susceptible to viral infection, which can easily lead to a large number of deaths. Therefore, to prevent significant economic losses, the spread of epidemics must be prevented via regional culling and disinfection. To promote the health of chickens, to protect the poultry industry, to provide consumers with safe poultry meat and eggs, and to reduce large-scale infection, chicks are vaccinated [2].

Chicks are mainly vaccinated against Marek disease (MD) [3], infectious bronchitis (IB), infectious bursal disease (IBD) [3], Newcastle disease (ND) [4], and other diseases. Different vaccines are administered using the corresponding vaccination methods at suitable locations. For example, needle-injected vaccines to chicks are mainly administered subcutaneously or intramuscularly [3]. Needle-injected vaccination is relatively difficult and can cause stress to chickens. With respect to MD, the etiologic pathogen is the herpes virus [5]. The virus easily infects chicks, and therefore, the Marek vaccine is injected subcutaneously into 1-day-old chicks [5].

For intra ovo injection, a hole is drilled into the eggshell at the top of the wide end of the egg, and then the injection needle is punctured into the egg membrane [1]. The inoculation locations for egg embryos vary among different vaccines [6,7]. The Marek vaccine shows the highest effectiveness when injected into the amnion [6,8]. Unlike the vaccination of chicks after the hatching of embryonic eggs, in ovo embryonic vaccine injection can stimulate the induction of embryos and enable chickens to develop immunity earlier [9]. The intra-egg injection is also used to inject nutrient solutions. In ovo feeding (IOF) early nutrition technology involves the injection of a nutrient solution into the amniotic fluid of late-stage embryonic eggs [10]; this solution is absorbed by the embryo along with the amniotic fluid [11], and it provides sufficient nutrition to the late embryos to protect them from starvation during the incubation period [12]. It can help overcome the limitations of embryonic development in eggs and nutrition in the late incubation period to improve the growth and development statuses of embryos, which is beneficial for subsequent growth.

However, this injection process causes stress to chicks, and consequently, these birds show difficulty in eating and a decrease in activity, which leads to a reduced survival rate. In ovo vaccination of embryonic eggs at approximately 18 days of age results in the best immune response, as chick immunity does not develop until the late embryonic stage [6,7,8,13,14,15]. At this time, the physiological structures of chick embryos are generally healthy, and vaccination at this time has minimal impact on the hatching rate of embryonic eggs [8]. At present, there are many commercial injection machines in the market for intra ovo vaccination, such as the Embrex Inovoject multi-egg injection machine (Zoetis, Inc., Parsippany, NJ, USA), Egginject^®^ Dual Pressure Injection System of Ceva Ecat-iD Campus, etc. After the completion of in ovo vaccination, the needles are sterilized before the next round of vaccine injection [16] to avoid cross-infection of embryos. Because of individual congenital differences, the structural position of each embryo is not precisely the same, and the location of the injection is also different [6]. The needle directly punctures the egg membrane during the injection process, and this may result in embryo injury affecting the hatchling survival rate and chick health. 

The needle-free technique has been widely used for the injection of vaccines or drugs in humans or mammals [17], and it can be injected repeatedly without the problem of cross-infection caused by incomplete needle disinfection [18]. The principle of needle-free injection involves mechanical compression to force a high-pressure fluid to penetrate the skin and subcutaneous tissue so as to deliver the drug by injection [18,19,20]. The purpose of this study was to establish a needle-free in ovo injection inoculation device for poultry eggs, which is electromagnetically driven to rapidly squeeze the plunger of the needle-free cartridge so that the high-pressure fluid in the cartridge is injected into the embryonic egg. A needle-free intra ovo injection device for poultry eggs was established in this study, and the positions and depths at which this injection could be administered under different jet pressures were determined. In addition, the changes and differences in the hatching rates of embryonic eggs after injection with the needle-free intra ovo injection device were observed.

## 2. Materials and Methods

The experimental protocol in the present study was approved by the National Chung Hsing University Animal Care and Use Committee (IACUC No.:108-081, Date of issue: 22 November 2019; IACUC No.:108-100, Date of issue: 14 January 2020).

This study involved four main experiments. In the first experiment, we used a self-developed electromagnetic-driven needle-free in ovo injection device to inject 18-day-old embryonic eggs with 0.25 mL blue pigment under different liquid jet pressures. The purpose of this experiment was to determine the depth and position where the injection fluid can be delivered in the embryonic egg at different jet pressures. In the second experiment, the stability of the needle-free in ovo injection device was measured at three jet pressures derived from the findings of the first experiment. In the third experiment, we measured the height of the air cell in 18-day-old embryo eggs and used a self-made electromagnetically driven needle-free in ovo injection device to test whether different air chamber heights affected the liquid jet pressure. Finally, in the fourth experiment, we observed the actual hatchability of 18-day-old Longmen embryonic eggs and specific pathogen-free (SPF) embryonic eggs after the injection of 0.25 mL 0.9% saline.

### 2.1. Sample

This study used 300 eggs with 18-day-old embryos of Lohmann variety and 140 eggs of specific pathogen-free with 18-day-old embryos. Specific pathogen-free chickens produced specific pathogen-free eggs. 

### 2.2. In Ovo Injection Device

**Figure 1:**
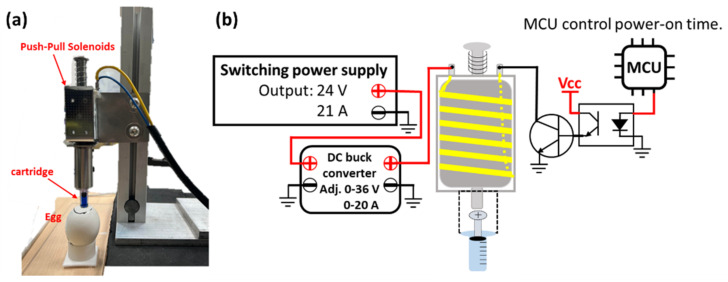
The injection device (**a**) Electromagnetic needle-free in ovo injection device (**b**) Circuit diagram of the electromagnetic needle-free in ovo injection device.

The needle-free in ovo injection device used in this study is shown in Figure 1a; it is electromagnetically driven, and the needle-free cartridge is squeezed at high speed by push-pull solenoids to inject liquids into embryonic eggs. The nozzle outlet of the cartridge vertically aligns with the center of the magnetic mounting base of the force sensor. The distance between the cartridge outlet and the magnetic base is 14 mm. A ZN63A push-pull solenoid (Liyuan Technology Co., Ltd., Zhuhai, China) was used, and it has a power-on time of 0.5 s/injection. The ejection force of the liquid in the cartridge is controlled by the ejector rod of the push-pull solenoid hitting the cartridge plunger. The greater the force of the rod impacting the cartridge plunger, the greater is the liquid jet pressure. A DC 24 V internal coil of the electromagnet was used. Therefore, the switching power supply powers the push-pull solenoid, and the maximum output voltage of the switching power supply is 24 V DC and 21 A. The switching power supply is controlled by a step-down module with constant voltage and constant current functions to supply these parameters to the push-pull solenoids. The adjustable output voltage and the current range of the step-down module are 0–36 V and 0–20 A, respectively, which control the pressure of the push-pull solenoid via the ejector rod, thereby controlling the cartridge liquid jet pressure. Therefore, the greater the input voltage and current of the push-pull solenoid, the stronger the magnetic field generated inside the push-pull solenoid, and the greater the impact force of the ejector rod. However, the coil will be heated and damaged if the push-pull solenoid is energized for too long. Therefore, the Atmaga328 single chip was used to control the power-on time of the push-pull solenoids, and the circuit structure is shown in Figure 1b. The push-pull solenoid changes resistance with an increase in the coil temperature due to continued use. Therefore, the research device adopts a constant current mode to supply power so that the magnetic force of the inner coil of the push-pull electromagnet is stable. When the coil was energized and excited, the elongation of the front-end rod was 33 mm, and the front end of the rod has a brass head with a diameter of 8 mm. After the electromagnet was excited, the plunger of the needle-free cartridge QS-P-03 (QS Medical Technology Co., Ltd., Beijing, China) was squeezed. The inner diameter of the upper cartridge where the plunger travels is approximately 5 mm, while the orifice diameter is 0.14 mm.

### 2.3. Measurement of Height of the Air Cell

Unlike needle-based injection, needle-free in ovo injection requires careful control of the liquid jet pressure of the device for the delivery of the injection fluid to the organs or site. Therefore, in this study, the height of the air chamber in 18-day-old Lohmann embryonic eggs was measured first, the purpose being the accurate measurement of the injection distance between the injection device and the egg. We also wanted to understand whether this distance change causes the injection jet pressure to decay when the liquid jet has not yet contacted the egg membrane of the air chamber during the injection process. The 18-day-old embryonic eggs were placed on the egg rack and a laser rangefinder was used to measure the distance perpendicular to the egg’s wide part (air cell end); then, after the marking of appropriate positions, holes with a diameter of 3 mm were drilled on the eggshell. The laser range finder measures the distance from the wide end to the membrane. The distance between the laser range finder and the egg perpendicular to the wide end of the eggshell was set as X, and that from the laser range finder to the air cell membrane was set as Y. The height of the air chamber (including the thickness of the eggshell) in 18-day-old embryonic eggs was the difference between X and Y.

On the basis of the three most commonly observed air cell heights, the distance between the force sensor and cartridge outlet of the needle-free injection device was adjusted. The liquid jet pressure was set to 7 MPa, and 30 injections were made at each height. The liquid in the injection cartridge was 0.25 mL of 0.9% saline, and the change in the liquid jet pressure at different injection heights was measured.

### 2.4. Process of Vaccination with the in Ovo Injection Device

**Figure 2:**
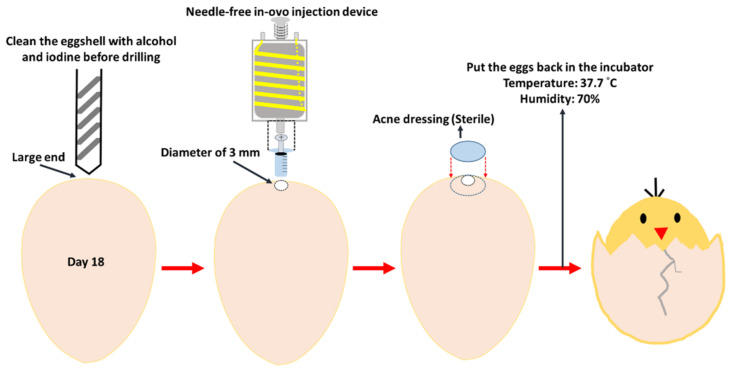
Needle-free in ovo injection procedure of embryonic eggs on day 18.

The embryonic eggs hatching process uses two hatchers (HY-01 and HY-03; Hoong Sheng Enterprise Co., Ltd., Changhua County, Taiwan) and the hatching capacity of the two hatchers is 126 and 252 eggs, respectively. Fertilized eggs were placed in the hatcher with a temperature of 37.7 °C and a humidity of 70%. The hatcher automatically turns the embryonic eggs in the hatcher from the first day of hatching. On the 18th embryonic day, the eggs were removed from the hatcher for the in ovo injection test. During the 18-day incubation process, all eggs were candled on day 9 of incubation; if the embryonic eggs died, were unfertilized, or stopped growing, they were removed from the hatcher to avoid contaminating the other eggs or affecting the normal growth of other embryonic eggs. For the needle-free intra ovo injection procedure, one egg tray (42 embryonic eggs) was taken out of the incubator for injection, at a time, and returned to the incubator after completion before removal of another egg tray. We removed embryonic eggs on day 18 from the hatcher and cleaned the shell where the holes were supposed to be drilled with alcohol and then the iodine. A hand-held electric drill was used to drill a hole approximately 3 mm in diameter at the wide end of the embryonic egg. After drawing 0.25 mL of 0.9% saline with the cartridge of the needleless syringe, the former was installed in the internal injection device. The injection was performed through the 3 mm wide hole in the eggshell. After the injection was completed, the hole was sealed with sterile acne dressing. Subsequently, the sealed embryonic eggs were placed back into the hatcher to allow them to grow. The embryo-egg injection processing flowchart is shown in Figure 2.

### 2.5. Measurement of Injection Depth According to the Jet Pressure

In this study, the needle-free injection was performed in the allantoic cavity and/or amniotic cavity of embryonic eggs. Therefore, the liquid injection pressure achieved by our needle-free in ovo injection device should be able to break through the inner shell membrane, but the injection pressure should not be too large to damage the embryo itself. The needle-free injection device was adjusted to 5, 6, 7, and 8 MPa of different liquid jet pressures to inject 0.25 mL of blue pigment. The 18-day-old embryonic eggs were injected with blue pigment to trace the relationship between the liquid jet pressure and injection depth.

### 2.6. Measurement of the Impact Pressure of the in Ovo Injection

The needle-free injection device must continuously inject the solution into different embryonic eggs. Therefore, the stability of the continuous injection jet pressure of the needle-free injection device was tested.

The liquid-jet impact pressure was measured using a quartz modal force sensor (9712B5, Kistler Instrumente, Winterthur, Switzerland) by administering 0.25 mL of 0.9% saline 30 times each at 6 MPa, 7 MPa, and 8 MPa. The maximum measuring limit of the force sensor was 5 lbf, and the sensitivity was 800 mV/lbf. The force sensor was placed under the orifice of the needleless injection device nozzle such that the liquid jet hits the center of the force sensor’s magnetic mounting base (Kistler, type: 800M159). The output voltage of the force sensor was captured using a frequency spectrum analyzer (SPIDER-20E, Magicdot Co., Ltd., Taiwan) and converted into a pressure value. Force measurements were performed at a rate of 8000 samples/s.

### 2.7. Measurement of Hatching Rates after Needle-Free in Ovo Injection

Hatching rates were observed to determine whether the needle-free in ovo injection procedure was correct and caused infection and other problems. The most important indicator for the success of needle-free in ovo injection in 18-day-old embryonic eggs is smooth hatching. Therefore, the subsequent hatching rate of embryonic eggs after injection with a needle-free injection device was measured in Lohmann embryonic eggs and specific pathogen-free embryonic eggs. The primary purpose was to verify whether the in ovo injection device established in this study could be used in embryonic eggs. The jet pressure of the needle-free injection device was adjusted to 6 MPa, 7 MPa, or 8 MPa for the injection of 0.9% saline (0.25 mL), and at each pressure, the injection was performed in 60 Lohmann eggs and 35 specific pathogen-free eggs. The corresponding control groups included 83 Lohmann eggs and 35 specific pathogen-free eggs without any treatment; the hatching survival rate was observed in the three pressure groups and the control group.

## 3. Results

### 3.1. Injection Depth According to the Jet Pressure 

**Figure 3:**
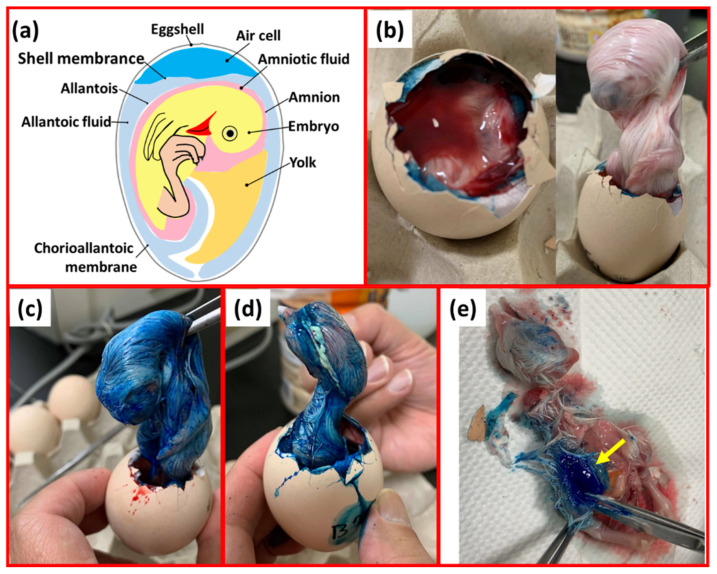
Simulated injection results (**a**) Spatial relations within an embryonated chicken egg; (**b**) 5 MPa needleless injection: the ink did not penetrate the inner egg membrane. (**c**) 6 MPa and (**d**) 7 MPa: blue pigment passed through the egg membrane, staining the surface of the chicken embryo. (**e**) 8 MPa: blue pigment has been injected into the muscle tissue of the chicken embryo.

As shown in Figure 3, the needle-free injection device was adjusted to 5, 6, 7, and 8 MPa of different liquid jet pressures to inject 0.25 mL of blue pigment. The 18-day-old embryonic eggs were injected with blue pigment to trace the relationship between the liquid jet pressure and injection depth.

### 3.2. Stability of the Jet Pressure 

The needle-free injection device must continuously inject the operation on different embryo eggs. Therefore, the stability of the continuous injection jet pressure of the needle-free injection device was simulated tested, as shown in Table 1.

### 3.3. Air-Cell Height 

**Figure 4:**
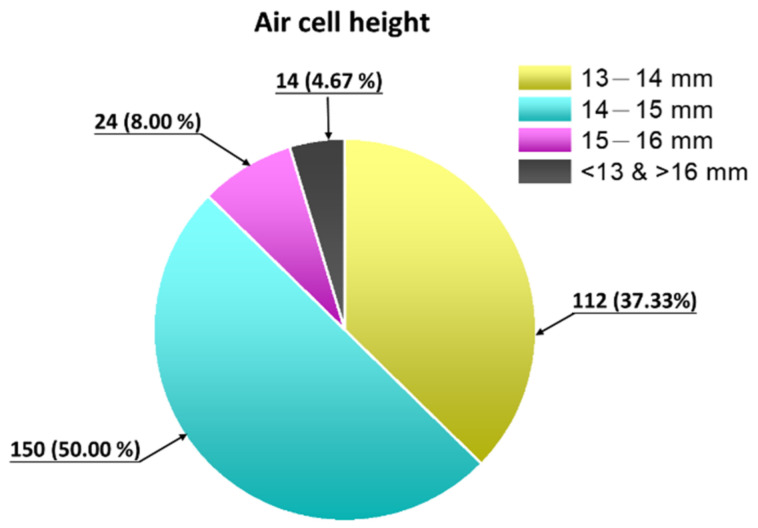
Height distribution map of air chambers in 300 18-day-old Lohmann eggs.

Figure 4 shows a total of 300 embryonic eggs of the 18-day-old Lohmann variety, where the height of the air chamber was 13–14 mm in 37.33% of eggs, 14–15 mm in 50.00% of eggs, 15–16 mm in 8.00% of eggs, and >16 mm or <12 mm in 4.67% of eggs.

As shown in Table 2, the nozzle of the needle-free injection cartridge was pressed against a 3 mm hole on the eggshell for needle-free injection with an average liquid jet pressure of approximately 7 MPa.

### 3.4. Hatching Rates after Needle-Free In-Ovo Injection

As shown in Table 3, the Lohmann eggs’ hatching rates were 100%, 98.3%, and 98.3% at 6 MPa, 7 MPa, and 8 MPa liquid injection pressures, respectively, while the control group was 100%. For the specific pathogen-free eggs, the hatching rates were 82.9%, 85.7%, and 68.6% at 6 MPa, 7 MPa, and 8 MPa, respectively; the control group was 85.7%.

## 4. Discussion

The needle-free injection device injected 0.25 mL of blue pigment into 18-day-old embryo eggs at pressures of 5, 6, 7, and 8 Mpa during initial injection trials. We found that the 5 MPa pressure was not sufficient for the penetration of the liquid from the egg membrane in the air cell into the allantoic and amniotic cavities and staining of the embryonic egg body (Figure 3b). However, 6 MPa and 7 MPa liquid injection pressures were sufficient for the liquid to reach the amniotic membrane and/or allantoic cavity through the egg membrane of the air chamber; the chicken embryo body was stained (Figure 3c,d). An injection pressure of 8 MPa was sufficient to inject the blue pigment into the muscle tissue of the chicken embryo (Figure 3e). However, because of the close proximity of the allantoic and amniotic cavities (Figure 3a), the current research method cannot precisely define the penetrative capacity of the liquid at a pressure of 6 or 7 MPa (amniotic cavity or allantoic cavity).

According to the results in Figure 3, the stability test was carried out on the injection pressure of the needle-free injection device at 6, 7, and 8 MPa as shown in Table 1. When the current was controlled at 7.4 Amps, 7.7 Amps, and 8 Amps, the average liquid jet pressures at settings of 6 MPa, 7 MPa, and 8 MPa were 6.31 MPa, 7.13 MPa, and 8.35 MPA with standard deviations of 0.24, 0.33, and 0.75, respectively. Therefore, the liquid jet pressure was relatively stable with a low level of variation. The settings of 6 MPa and 7 MPa yielded relatively accurate average jet pressures. Therefore, when these settings were used (Figure 3c,d), the injected liquid could reach the amniotic cavity and/or allantoic cavity through the egg membrane to stain the embryo body. An injection pressure of 8 MPa was sufficient to inject the liquid into the muscle tissue of the chicken embryo (Figure 3e).

Therefore, 300 18-day-old embryonic eggs were measured for the air cell height. The purpose was to understand whether changes in the height of the air chamber during the injection process could affect the injection pressure. From Figure 4, the height of the air chamber was 13–14 mm in 37.33% of eggs and >16 mm or <12 mm in 4.67% of eggs. Therefore, attenuation of liquid jet pressure was measured at distances of 13 mm, 14 mm, and 15 mm.

We found that a 13–15 mm height does not cause the attenuation of the liquid jet pressure, and the average liquid jet pressure was approximately 7 MPa, as shown in Table 2. Therefore, in subsequent experiments, when injecting liquids into embryonic eggs, the nozzle of the needle-free injection cartridge was pressed against a 3 mm hole on the eggshell for needle-free injection.

In ovo needle-free injection experiments were performed using two types of 18-day-old embryonic eggs, 263 Lohmann breeder eggs and 140 specific pathogen-free eggs, respectively. Hatching rates were observed to determine whether the needle-free in ovo injection procedure was correct and caused infection and test injection device real availability and other problems.

As shown in Table 3, the hatching rates were 100%, 98.3%, 98.3% at 6 MPa, 7 MPa, and 8 MPa, and that in the control group was 100%. One death each occurred at liquid jet pressures of 7 MPa and 8 MPa. The anatomy revealed that the two embryonic eggs had been developing relatively slowly, and neither had fully developed into 18-day-old embryonic eggs. Therefore, premature injection resulted in death because the developmental time was insufficient. With respect to the specific pathogen-free eggs, the hatching rates were 82.9%, 85.7%, and 68.6% at 6 MPa, 7 MPa, and 8 MPa, respectively, and that in the control group was 85.7%. Specific pathogen-free eggs, which are guaranteed to be free from specific pathogens, are specially used for vaccine production and biological research. They are more susceptible to pathogen infection and death during incubation than are ordinary embryonic eggs. As shown in Table 3, the hatching rate of specific pathogen-free eggs after saline injection at 6 MPa and 7 MPa was not significantly different from that in the control group. Injecting fluid into the allantoic cavity and/or amniotic cavity of embryonic eggs at 6 MPa and 7 MPa did not affect the hatchability of chicks. However, it must be noted that cleaning the eggshell with alcohol and iodine before drilling the hole and sealing the hole with sterile acne dressing prevented infections and consequent deaths in the embryonic egg. The hatching rate of specific pathogen-free eggs after 8 MPa saline injection was only 68.6%. From the previous ink injection experiments, it can be inferred that at the injection pressure of 8 MPa, the injected liquid could reach the embryo. Therefore, the hatching rate may have been reduced because of embryo injury. Both Lohmann eggs and specific pathogen-free eggs showed excellent hatching performance after needle-free injection. The hatchability of needle-free intra ovo injections in this paper was comparable to that found by Zhai et al. [21], who used the needle injection method to administer 100-μL of 0.85% sterilized saline solution into the amnion of 18- and 17-day-old White Leghorns embryonic eggs. Among them, the hatching rate of those injected with saline solution at 18 days old was 94%, whereas that resulting from injection at 17 days old was 74%. In addition, a group of eggs that received no injection was used as the control group, and the hatchability of the 18-day-old group was 94%, and that of the 17-day-old group was 76% [21]. Moreover, McGruder et al. [22] used the IntelliLab single egg injector (AviTech LLC, Salisbury, MD, USA) to inject 200 μL of 117 mM saline solution into the amnion of 18-day-old Broiler hatching eggs (Ross × Ross 308), obtaining a hatching rate of 88.5%, whereas the hatchability of the respective control group without injection was 90.8% [22]. The in ovo needle-free injection device described herein is therefore comparable to in ovo injection using needles or other commercial injection devices described in the literature. Furthermore, in the present study, all the injections performed during the experiment contained saline solution, and there was no significant difference between with other studies in the final hatching rate. In summary, injections of 0.25 mL of 0.9% saline into embryonated eggs at pressures of 6 MPa and 7 MPa were feasible. The injected liquid was able to reach into the allantoic cavity and/or the amniotic cavity of the embryonic egg at 6 MPa, and 7MPa injection pressure did not affect the hatchability of chicks. In the future, the needle-free injection device in this paper has the potential to replace vaccines with saline such as MD or nutrient solution. The injection vaccines or nutrients [10,21] could be delivered to the allantoic cavity and/or amniotic cavity with this method.

## 5. Conclusions

The electromagnetic needle-free in ovo injection device established in this study can inject 0.25 mL of saline liquid into the allantoic cavity and/or amniotic cavity of 18-day-old embryonic eggs at jet pressures of 6 MPa and 7 MPa. At 8 MPa injection pressure, saline was injected into the muscle tissues of embryonic eggs. The hatching rate of saline-injected Lohmann eggs was 98.3% and that of the specific pathogen-free eggs was 85.7% at 7 MPa pressure. The application of the electromagnetic in ovo needle-free injection device established in this study has been tested in commercial hatching eggs and specific pathogen-free eggs specially used for vaccine production and biological research. As an actual application of the needle-free in ovo injection device, the saline can be replaced with actual vaccines of diseases such as MD or ND, and the liquid jet pressure can be adjusted according to the target embryonic tissue. Needle-free in ovo injection can help avoid traditional needle-type in ovo vaccination, embryo injury caused by injection needle puncture, or cross-infection caused by unclean needles, prevent replacement needle and needle cleaning steps, and effectively improve animal welfare. In addition, this study can serve as a reference for future studies on needle-free in ovo injection automation systems, jet control, and injection pre-processing procedures.

## Figures and Tables

**Table 1:** Average liquid jet pressure and device control of the needle-free in ovo injection device at 6–8 MPa.

Performance of Targeted Injection Pressure	Setting Liquid Injection Pressure (MPa)		
	6	7	8
Average liquid injection pressure (MPa)	6.31	7.13	8.35
Standard Deviation (MPa)	0.24	0.33	0.75
Current limiting (A)	7.4	7.7	8
0.25 mL of 0.9% saline, Injection distance: 14 mm			

**Table 2:** Variation in the liquid jet pressure of ~7 MPa at different heights.

Distance from Cartridge Outlet to the Inner Membrane of the Air Cell in the Egg (mm)			
	13	14	15
Average liquid injection pressure (MPa)	7.06	7.13	7.10
Standard deviation (MPa)	0.21	0.19	0.18

**Table 3:** Hatching rates of Lohmann and specific pathogen-free embryonic eggs injected with saline at different liquid jet pressures.

**Lohmann Breeder Eggs**				
Liquid injection pressure (MPa)	6	7	8	Control group
Hatchability (%)	100 (60/60)	98.3 (59/60)	98.3 (59/60)	100 (83/83)
**Specific Pathogen-Free Eggs**				
Liquid injection pressure (MPa)	6	7	8	Control group
Hatchability (%)	82.9 (29/35)	85.7 (30/35)	68.6 (24/35)	85.7 (30/35)

## Data Availability

The data presented in this study are available on request from the corresponding author.
